# Fatty acid metabolism: A new therapeutic target for cervical cancer

**DOI:** 10.3389/fonc.2023.1111778

**Published:** 2023-03-28

**Authors:** Pengbin Ping, Juan Li, Hongbin Lei, Xiaoying Xu

**Affiliations:** Department of Radiotherapy Oncology, The Second Affiliated Hospital of Dalian Medical University, Dalian, China

**Keywords:** cervical cancer, fatty acids, fatty acid metabolism, metabolic reprogramming, therapeutic target

## Abstract

Cervical cancer (CC) is one of the most common malignancies in women. Cancer cells can use metabolic reprogramming to produce macromolecules and ATP needed to sustain cell growth, division and survival. Recent evidence suggests that fatty acid metabolism and its related lipid metabolic pathways are closely related to the malignant progression of CC. In particular, it involves the synthesis, uptake, activation, oxidation, and transport of fatty acids. Similarly, more and more attention has been paid to the effects of intracellular lipolysis, transcriptional regulatory factors, other lipid metabolic pathways and diet on CC. This study reviews the latest evidence of the link between fatty acid metabolism and CC; it not only reveals its core mechanism but also discusses promising targeted drugs for fatty acid metabolism. This study on the complex relationship between carcinogenic signals and fatty acid metabolism suggests that fatty acid metabolism will become a new therapeutic target in CC.

## 1 Introduction

Cervical cancer (CC) seriously affects women’s life and health. Globally, approximately 530,000 new cases and 266,000 new deaths are reported annually (1). Early HPV detection and vaccination have greatly reduced the incidence of CC and grade 3 cervical intraepithelial neoplasia (CIN3) in young women (2). Even though CC can be actively prevented, it still remains the second leading cause of cancer-related death among young and middle-aged women (3). The most common histological subtype of CC is cervical squamous cell carcinoma (CSCC) (4), which accounts for approximately 70% of all CC cases in the United States (5) and approximately 90% of CC cases in China (6, 7). SCC Ag is considered to be the most clinically valuable serum tumor marker of SCC, and its levels are usually associated with larger primary tumors, later stage, and lymph node involvement (8–10). Of note, nearly a quarter of CC patients do not have elevated SCC Ag levels (11). Furthermore, almost all CCs are related to high-risk human papillomavirus (HPV) infections, of which nearly 50% are HPV16 infections (12, 13). However, HPV infection is not a requirement. Approximately 3-8% of cases are HPV-negative CC (14–16). The American Joint Committee on Cancer (AJCC) 9th edition TNM CC staging, based on histopathological observations, was updated to reflect HPV-associated and HPV-unrelated carcinomas. Moreover, HPV-independent cervical cancer is usually linked to early lymph node metastasis and is more often diagnosed with nonsquamous histology (17, 18). Interestingly, HPV infection does not always develop into cancer; there must be additional changes. At present, most patients diagnosed with advanced CC miss the opportunity for radical surgery. The standard therapy for locally advanced cervical cancer (LACC) is concurrent chemoradiotherapy (CCRT) (19). However, the prognosis of stage III and IV patients remains poor, with 5-year progression-free survival (PFS) and overall survival (OS) rates of 51% and 55%, respectively (20). Pelvic lymph node metastasis remains an important independent prognostic factor for CC In addition, it is associated with a lower 5-year survival rate and a higher recurrence rate (21–23). However, there is no effective method to control and prevent lymph node metastasis. Although the use of targeted and immunological agents has improved survival to some extent (24, 25), there is still an unmet need for additional treatment for patients with node-positive and recurrent CC. Therefore, it is necessary to explore novel and promising therapeutic targets for CC.

As early as 1956, Otto Warburg found that glucose metabolism differed substantially between normal cells and cancer cells. Even when oxygen is abundant, cancer cells preferentially convert pyruvate to lactate rather than utilizing glucose to produce maximum energy; thus, glucose consumption increases (26). Recently, increasing studies have revealed the metabolic kinetics of cancer and subsequently introduced the concept of metabolic plasticity or metabolic recombination of cancer cells. In addition to the use of glucose, cancer cells undergo a variety of carcinogenic mutations or adaptations to allow the use of more diverse nutrients, including fatty acids, to promote tumor survival, metastasis and disease progression (27). These research achievements have led to renewed interest in defining the various roles of lipid metabolism in cancer. Moreover, the dysregulation of fatty acids and related lipid metabolism pathways can affect the occurrence of a variety of malignant tumors and lead to poor prognosis (28, 29). Interestingly, in previous studies, CC was considered to be related to lipid metabolism, which can promote the occurrence and development of cervical cancer to a certain extent (30, 31).

**Figure 1:**
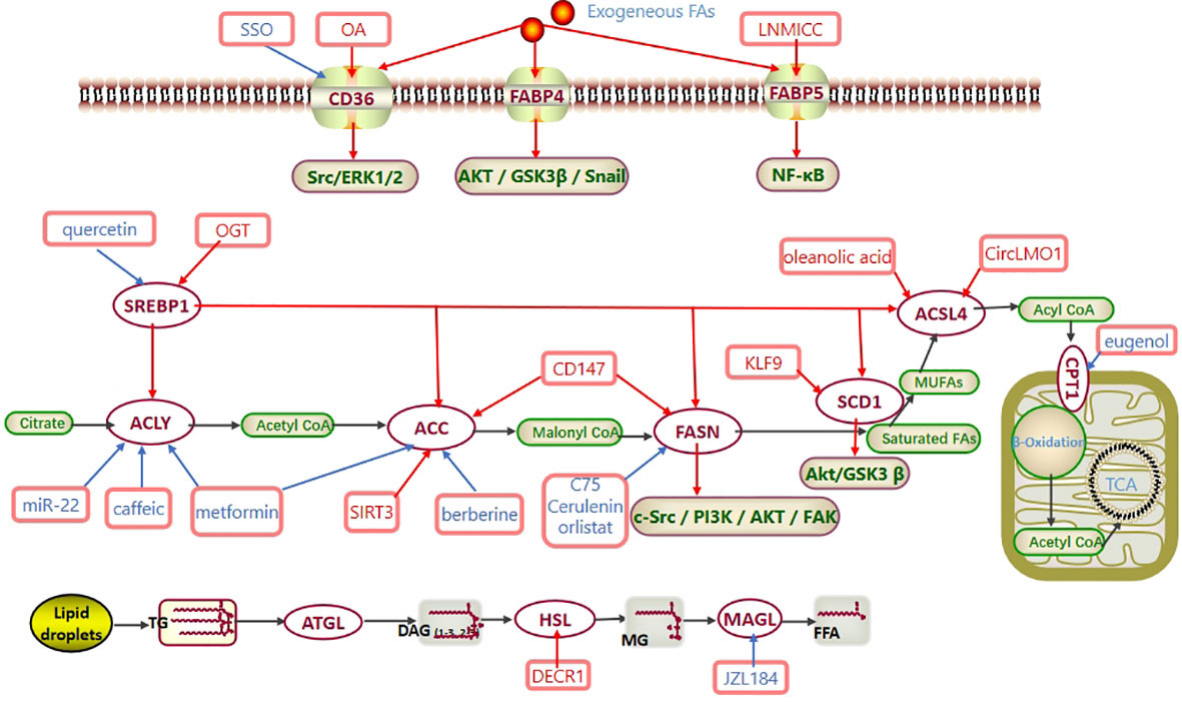
Fatty acid metabolism in CC. Exogenous fatty acids are ingested through CD36 and FABP. SREBP1 regulates the expression of ACLY, ACC, FASN, SCD1 and ACSL4 at the transcriptional level. Activated acyl-CoA enters mitochondria *via* CPT to participate in β-oxidation and generate acetyl-CoA. Eventually, acetyl-CoA enters the TCA cycle to produce ATP. In CC, CD36 promotes the Src/ERK1/2 pathway, FABP4 promotes the AKT/GSK3β/Snail pathway, FABP5 promotes the NF-κB pathway, FASN promotes the c-Src/PI3K/AKT/FAK pathway, and SCD1 promotes the Akt/GSK3β pathway to regulate the progression of cervical cancer. Abbreviations: TCA cycle, tricarboxylic acid cycle; CD36, cluster of differentiation 36; FABP, FA-binding protein; SREBP1, sterol regulatory element-binding protein 1; ACLY, ATP–citrate lyase; ACC, acetyl-CoA carboxylase; FASN, fatty acid synthase; SCD1, stearoyl-CoA desaturase-1; MUFAs, monounsaturated fatty acids; ACSL4, long-chain acyl-CoA synthetases 4; CPT1, carnitine palmitoyltransferase 1; ATGL, adipose triglyceride lipase; HSL, hormone-sensitive lipase; MAGL, monoglyceride lipase; SSO, an inhibitor of CD36; OA, oleic acid; OGT, O-linked N-acetylglucosamine transferase; C75, cerulenin, orlistat, the inhibitors of FASN; eugenol, an inhibitor of CPT1; JZL184, an inhibitor of MAGL.

This review aimed to better understand the roles of fatty acid metabolism and related lipid metabolism pathways in CC. We reviewed a large number of studies, examined the effects of various fatty acid metabolic pathways in CC, and focused on their mechanisms of action and future perspectives (**Figure 1** and **Table 1**).

**Table 1:** Role of fatty acid metabolism and related lipid metabolism pathways in cervical cancer (CC).

FA Metabolism	Target	Effects and Features	References
FA Synthesis	FASN	FASN promoted CC cell migration, invasion, and lymphangiogenesis	(28, 32, 33)
	ACLY	MiR22 downregulated ACLY and attenuated CC cell proliferation and invasion	(34)
	ACC	Silencing of ACCα significantly promoted the apoptosis of CC cells	(35–37)
	SCD1	SCD1 level was associated with the CC stage, the overall survival rate, and the disease-free survival rate	(38)
FA Uptake	CD36	Overexpression of CD36 promoted the invasion and metastasis of CC cells *in vitro* and *in vivo*	(39–42)
FA Transport	FABP	FABP promoted epithelial-mesenchymal transition, lymphangiogenesis, and LNM by reprogramming fatty acid metabolism	(43–47)
FA Activation	ACSL4	Upregulated ACSL4 expression promoted CC cells ferroptosis	(48–50)
FA Oxidation	CPT1A	High expression of CPT1A promoted lipid metabolism modification and CC progress	(51)
Intracellular Lipolysis	ATGL		(52)
	HSL	Enhanced lipid catabolism contributes to the malignant progression of CC	(53)
	MAGL		(54)
Transcription Factors Lipid Metabolism	SREBP LPA CHOL	High expression of SREBP-1 promoted the proliferation of CC cells LPA inhibited apoptosis of CC cells induced by chemotherapy Intersection of different metabolic pathways in CC	(55, 56) (57, 58) (28, 59)

## 2 Targeting *de novo* fatty acid synthesis

A well-studied aspect of cancer metabolism is the upregulation of *de novo* fatty acid synthesis (60). Unlike normal cells, tumor cells shift lipid acquisition from fatty acid uptake to enhanced *de novo* fatty acid synthesis to acquire characteristics of unlimited proliferation, thus providing survival advantages for tumor cells and inducing tolerance to radiotherapy and chemotherapy (61, 62). The production of nascent fatty acids is mediated by a variety of enzymes, including fatty acid synthase (FASN), stearoyl-CoA desaturase 1 (SCD1), ATP-citrate lyase (ACLY), and acetyl-CoA carboxylase (ACC). Here, we study their respective mechanisms of action in CC.

### 2.1 Fatty acid synthase (FASN)

Enhanced expression of fatty acid synthesis enzymes is one of the important metabolic adaptations in some malignant tumors (60, 63). For example, free fatty acids promote the proliferation and invasiveness of estrogen receptor alpha-positive breast cancer cells by activating the mTOR pathway (64). In addition, studies have shown that elevated fatty acid synthesis levels may be an early metabolic deregulation in Myc-driven prostate cancer (63). Xu et al. demonstrated that plasma fatty acid composition levels are highly likely to be potential biomarkers for ovarian cancer and other gynecological tumors (65, 66). Carcinogenic transformation is closely related to fatty acid metabolism (67). Because it provides a large amount of energy for the proliferation of malignant tumors, FASN, as the hub of lipid metabolism, plays an increasingly prominent role in tumors with lipid-rich phenotypes. In fact, a series of new FASN inhibitors have been designed. Interestingly, an experiment confirmed that the expression of FASN was upregulated in patients with CC and was associated with lymph node metastasis (28). Additionally, the correlation between the expression of FASN and clinicopathological features was evaluated, and FASN was identified as an independent prognostic factor in patients with CC by multivariate Cox proportional hazard analysis. The specific mechanism involves the ability of FASN to regulate cholesterol reprogramming, which leads to disordered lipid raft-related c-Src/PI3K/AKT/FAK signaling and further increases the invasiveness of CC cells (28). This mechanism has also been elucidated in ovarian cancer (68, 69). In addition, FASN induces lymphangiogenesis through the production of PDGF-AA/IGFBP3.Meanwhile, the FASN inhibitors cerulenin and C75 can significantly inhibit lymph node metastasis of CC (28). Although cerulenin has effects on various types of tumors *in vitro* and *in vivo*, the high activity and off-target activity of cysteine reactive epoxides hinder their clinical development, and the side effects of cerulenin and C75 in mice also include serious weight losses (70, 71). Another FASN inhibitor that has been widely investigated is orlistat, which is an anti-obesity drug approved by the FDA that has been proven to be effective in tumor biology (72, 73). Interestingly, a study demonstrated that the expression of FASN in CC was higher than that in cervical benign lesions and increased with the increase of the disease stage; however, statistical results showed no significant correlation between the expression of FASN and the grade of cervical lesions. However, in cell experiments, orlistat significantly inhibited the growth and proliferation of CC cells, and this effect was more significant in HPV16-positive and HPV18-positive CC cells. The mechanism underlying these effects was not related to necrosis but was related to apoptosis (32). One of the reasons for this result is that the sample size of this study was relatively small, and it may also be possible that FASN plays an important role in early tumor transformation. However, previous studies have shown that the expression of FASN is related to epithelial-mesenchymal transformation (EMT). FASN can promote EMT in breast cancer, while inhibition of FASN can reverse the EMT process (74). These results are compatible with those of other studies showing that the FASN inhibitor orlistat inhibits CC cell proliferation and blocks lymph node metastasis (30, 33). However, oral orlistat can cause significant gastrointestinal dysfunction, such as fat leakage and abdominal distention (75). Considering the important role of FASN inhibitors in cancer, researchers designed new protocols, such as nanoencapsulation, to improve their oral bioavailability and solubility (76).

To overcome the shortcomings of first-generation FASN inhibitors, researchers have focused on designing FASN inhibitors with superior selectivity, reversibility and nonreactivity to improve drug performance. For example, JNJ-54302833, GSK2194069, IPI-9119, and TVB-2640 have been developed, but only TVB-2640 was used in clinical trials (77). Moreover, treatment with TVB-2640 has shown potent effects in all kinds of solid tumors, including breast cancer, KRAS-mutated non-small cell lung cancer, and CC, and the combination of TVB-2640 and paclitaxel has shown effective target binding (78). Moreover, TVB-2640 is well tolerated, and adverse effects can be reversed when the drug is discontinued (79). A clinical trial involving TVB-2640 in combination with paclitaxel and trastuzumab in HER2-positive advanced breast cancer is under evaluation (80).

Certainly, FASN inhibitors are not universal, there are differences in sensitivity to different metabolic inhibitors and in metabolic characteristics of different tumors and different subtypes of the same tumor. Therefore, it is necessary to clearly understand the metabolic susceptibility of different tumors. After metabolite analysis, pancreatic cancer cells can be divided into three subtypes, namely, the low-proliferative subtype, the lipogenic subtype and the glycolytic subtype. Only the lipogenic subtype shows good sensitivity to FASN inhibitors, indicating the plasticity of the metabolic network of cancer cells (81). Therefore, to accurately predict the sensitivity of cancer cells to FASN inhibitors, we first need to understand the interactions between the different metabolic networks of cancer cells.

### 2.2 ATP-citrate lyase (ACLY)

ACLY catalyzes the conversion of citrate to oxaloacetate and acetyl-CoA. A variety of ACLY inhibitors have been used to treat hyperlipidemia (82, 83). ACLY is a key enzyme linking fatty acid synthesis with glycolysis. ACLY has been shown to be highly expressed in a variety of cancers (84), and its inhibitor has a more significant anticancer effect in high-glycolytic cells (85). The PI3K/Akt pathway plays an important role in CC (86). Hyperglycolysis promotes tumor growth by increasing ACLY levels and fatty acid synthesis through the activation of PI3K/Akt signaling (85). Caffeic acid combined with metformin downregulates the expression of the ACLY protein by activating AMPK, which further reduces fatty acid synthesis, resulting in an increase in the apoptosis rate of metastatic cervical HTB-34 cells (87). Mei et al. showed that the level of ACLY was increased in CC cells. Furthermore, miR-22 could mediate the downregulation of ACLY and accelerate the apoptosis of CC cells. It was also found that the tumor weight in mice treated with miR-22 was much lower than that in the control group. The mechanism underlying these effects may be that miR-22 reduced the ability of *de novo* lipid synthesis by inhibiting the expression of ACLY, thus inhibiting the proliferation and invasion of cancer cells (34). The combination of an AMPK activator and an ACLY inhibitor may be another strategy for cancer treatment (88). There are few studies on targeting ACLY in the treatment of CC, but it is undeniable that it may be a powerful potential target for the treatment of CC.

### 2.3 Acetyl-CoA carboxylase (ACC)

ACC is a rate-limiting enzyme that catalyzes the formation of malonyl-CoA from acetyl-CoA. There are two subtypes of mammalian ACC, ACCα (ACC1 or ACACA) and Accβ (ACC2 or ACACB) (89). While ACCα is enriched in adipose tissue (90), ACCβ mainly exists in oxidized tissues (91). These expression patterns determine the difference in its metabolism in different tissues. Gu et al. performed immunohistochemical staining of CD147, a transmembrane glycoprotein, in 85 cases of CC and 24 cases of normal cervical epithelia. CD147 was highly expressed in CC, with a positive rate of 78.7%. *In vitro* experiments showed that CD147 could promote the proliferation and lymph node metastasis of CC cells. The mechanism involves the reprogramming of lipid metabolism by CD147 through FAS and ACC1. After CD147 knockdown, the lipid content of CC cells was markedly reduced, and the migration ability of cancer cells was also greatly reduced (33). Li et al. reported that SIRT3 could accelerate lipid synthesis by upregulating ACCα in CC oncogenic tissues, thereby increasing their invasion. Moreover, in an allograft mouse model, the tumors were significantly larger in the high SIRT3 expression group than in the SIRT3 knockout group (35). Consistent with this finding, studies have shown that the level of ACACA is upregulated in CC cells. Silencing ACACA can accelerate the apoptosis of CC cells (36). Previous studies have shown that berberine can inhibit the proliferation of CC cells by reducing the activity of ACC and the synthesis of intracellular fatty acids, resulting in the decreased production of extracellular vesicles (37). Similarly, metformin can activate AMPK and downregulate ACCα levels in CC cells to inhibit lipid synthesis, thereby inhibiting tumor growth (87). Interestingly, silencing ACCα or ACCβ can promote NADPH-dependent redox balance, leading to the accelerated growth of lung cancer cells (92). At the same time, ACC levels may be useful for predicting the prognosis of some patients undergoing anticancer treatment. A MITO phase III trial found that the increase in the phosphorylation level of ACC predicted poor outcomes in patients with ovarian cancer treated with paclitaxel/carboplatin (93). The role of ACC in tumors is complex, but these indicate that targeting ACC is a potential therapeutic strategy for CC. However, to date, no ACC inhibitor has reached the stage of clinical trials for gynecological cancers.

### 2.4 Stearoyl-CoA desaturase-1 (SCD1)

The transformation of saturated fatty acids to monounsaturated fatty acids requires the catalysis by SCD1, which can promote the occurrence of a variety of tumors and accelerate their malignant progression. However, cancer progression may result from an imbalance between unsaturated and saturated fatty acids (94, 95). The expression level of SCD1 is highly upregulated in several malignancies, such as ovarian (96, 97), gastric (98), and lung cancer (99), and is associated with poor prognosis. SCD1 targeting or gene knockout can significantly inhibit tumor growth and restore cisplatin resistance (99–101). Wang et al. analyzed the role of SCD1 in CC using the GEPIA database. A total of 306 CC samples and 13 normal samples were included. It was found that the expression levels of SCD1 in CC tissues were high and were related to the overall survival time and staging of patients. At the same time, the low expression of KLF9 was found in advanced CC and negatively correlated with that of SCD1. The final results showed that the expression of KLF9/SCD1 could regulate the Akt/GSK3-β signaling pathway in CC cells and affect the proliferation, invasion and EMT process of CC cells. This phenomenon can be suppressed by knocking out SCD1 (38). Studies have also shown that SCD1 can regulate the level of miR-1908, and its high expression levels can promote the proliferation and invasion of CC cells (59, 102). Interestingly, a study has shown that metformin can downregulate SCD1 expression, thereby inhibiting CC cells (87). Notably, SCD1 protects tumor cells from ferroptosis (103). Therefore, targeting SCD1 provides a new idea for the treatment of CC. To date, however, virtually no SCD1 inhibitors have been clinically tested as cancer therapies in humans.

## 3 Targeting fatty acid uptake

The growth of tumor cells depends on the intake of exogenous fatty acids, which can promote tumor progression and metastasis (104). The intake of exogenic fatty acids mainly depends on FABP, LDLR, and CD36, which is a member of the FATP family (105). CD36 is negatively linked to the prognosis of patients and is an important biomarker of malignant tumors (106). Previous studies have demonstrated that CD36 levels are upregulated in some malignancies, such as ovarian cancer (104), breast cancer (107), gastric cancer (108), oral cancer (109), melanoma (110), and colorectal cancer (111), to maintain cancer cell progression and metastasis. In one experiment, 133 cases of CC and 47 cases of normal cervical tissues were evaluated. In normal cervical tissues, CD36 expression was detected only in 19.15% of tissues (9/47), while in CC cases, CD36 immunoreactivity was detected in 73.68% of tissues (98/133). The evaluation results showed that CD36 was closely associated with CC progression. High CD36 levels are associated with enhanced EMT, tumor differentiation and lymph node metastasis through synergistic interactions with TGF-β (39). Another study showed that dietary oleic acid, was linked with an increase in malignant tumors in HeLa cells. Oleic acid induced the activation of Src kinase and the downstream ERK1/2 pathway in a CD36-dependent manner, and the overexpression of CD36 in HeLa cells aggravated tumor growth and invasion in xenograft mice. The CD36 inhibitor sulfonyl-n-succinic acid oleic acid (SSO) can specifically and irreversibly bind to CD36 and reverse the process of malignant transformation by inhibiting the uptake of fatty acids (40). Increasing expression level of miR-1254 could inhibit the invasion of SiHa and CaSki cells. Additionally, the increase in the expression of CD36 significantly enhanced the proliferation of CC cells, and the increase in the expression of CD36 reversed the inhibitory effect of miR-1254 (41). Similarly, An et al. confirmed that the expression of CD36 in CSCC tissues was higher than that in normal cervical tissues, and the change in CD36 expression level was a unique feature associated with HR HPV infection. HR HPV infections could promote the tumorigenesis and progression of CC and are associated with shorter recurrence-free survival (42). In conclusion, we predict that CD36 is a breakthrough target for the treatment of CC.

## 4 Targeting fatty acid activation

Fatty acids need to be converted into acyl-CoA to be activated before lipid synthesis and oxidation. The enzyme mediating this process is long-chain acyl-CoA synthetase (ACSL), which can activate the most abundant long-chain fatty acids (112, 113). There are 5 subtypes of ACSL in mammals (ACSL1, ACSL3, ACSL4, ACSL5 and ACSL6), each with specific functions. Among them, ACSL4 is the best studied. ACSL4 can promote uncontrolled cell growth and enhance tumor escape from programmed cell death and invasion (112, 114, 115). ACSL4 is highly expressed in ovarian (116), prostate (113), liver (117), breast (118) and other tumors and is associated with poor prognosis. Interestingly, oleanolic acid (OA), which is naturally present in plant fruits and leaves, enables dramatic inhibition of the mass and volume of CC tumors in mice, and ACSL4 expression remains highly upregulated in CC cells and xenograft models treated with OA. When the level of ACSL4 is inhibited by siRNA, OA no longer has the ability to inhibit cancer cells (48). The mechanism underlying these effects may be that OA promotes ferroptosis by upregulating ACSL4 levels. Circular RNA (circRNA) has been shown to limit the progression of malignant tumors. Circular RNA (CircLMO1) has been demonstrated to promote ferroptosis induced by the high expression of ACSL4 in CC cells and prevent the growth and invasion. Additionally, ACSL4 knockdown abolished the inhibitory effect of CircLMO1 on CC cells (49). Zhao et al. demonstrated that ACSL4-mediated ferroptosis plays an essential role in the effect of the combination of paclitaxel and propofol against cancer (50). More interestingly, Li et al. demonstrated that the expression of ACSL4 was significantly lower in patients with lung adenocarcinoma, and the prognosis was poor compared with that in patients with high expression of ACSL4 (119). By contrast, some studies have also proven that the high expression of ACSL4 promotes the development of lung cancer. Owing to the heterogeneity of tumors, the role of ASCL4 in different cancers is not consistent, and the mechanism of ACSL4 in cancer promotion and inhibition is complex and variable. However, targeting ACSL4 can regulate tumor progression, so ACSL4 is very likely to be a novel target for treatment.

## 5 Targeting fatty acid oxidation

Fatty acid oxidation (FAO) must first occur through the action of carnitine palmitoyl transferase (CPT), which is composed of CPT1 and CPT2 located in the outer and inner membranes of mitochondria. CPT1 has three isoforms, CPT1A, CPT1B and CPT1C (120, 121). Recently, FAO has been suggested to be closely linked to cancer progression, proliferation and drug resistance. ATP is significantly decreased by blocking FAO in cancer cells (121–123). CPT1A is highly expressed in prostate cancer (124), nasopharyngeal carcinoma (125, 126), glioblastoma (127), etc. Inhibition of CPT1A significantly inhibits tumor growth, improves survival, and increases the sensitivity of nasopharyngeal carcinoma to radiotherapy (125). Almost all CCs are associated with high-risk HPV infections, and HPV16 is responsible for nearly 50% of infections (12, 13). The level of CPT1A in HPV16-positive CC tissues was reported to be markedly higher than that in normal tissues, suggesting that CPT1A could promote cervical cancer progression through lipid metabolism modifications (51). The overexpression of adipose-triglyceride lipase (ATGL) was also found to be dependent on the induction of hypoxia-inducible factor-1α (HIF1α) by reactive oxygen species (ROS), which are mediated by increased mitochondrial FAO to promote CC cell proliferation (52). Xiao et al. demonstrated that SIRT3 can promote the invasion of CC cells by activating the AMPK/PPAR pathway (128). Notably, AMPK activators and PPAR activators can induce CPT1 expression, thereby enhancing FAO (129, 130). In a phase III clinical trial conducted in a resource-scarce environment, eugenol, a CPT1 inhibitor, as one of the ingredients of antiviral AV2, was slightly effective in inducing the regression of cervical precancerous lesions, although there was no statistically significant difference between the treatment and placebo groups. However, this lack of statistical significance may change in a later stage in a high-resource environment and by expanding the sample size (131). Research on the involvement of CPT in cervical cancer seems to have received little attention, but it may bring new insights into the treatment of CC.

## 6 Targeting the intracellular transport of fatty acids

Fatty acid-binding proteins (FABPs) are a series of lipid chaperones and members of the superfamily of intracellular lipid-binding proteins. These proteins are mainly involved in the transport of intracellular fatty acids between organelles and promote fatty acid solubilization and metabolism. Recent studies have found that FABPs play an increasingly prominent role in oncology, and tumor progression and invasion may be linked to an elevated level of an exogenous FABP (132). A study has shown that circulating levels of A-FABP, also known as FABP4, are significantly higher in obese patients with breast cancer than in those without breast cancer, and circulating A-FABP enhances tumor stemness and aggressiveness by activating the IL-6/STAT3/ALDH1 pathway (133). FABPs are found not only in breast cancer but also in ovarian cancer (134, 135), acute myeloid leukemia (136), and liver cancer (137, 138). Interestingly, both FABP4 and FABP5 seem to play a role in CC. Real-time quantitative PCR and western blotting were used to evaluate the expression of FABP5 in 206 CC and 40 normal cervical tissues, and the mRNA and protein expression of the FABP5 was found to be significantly upregulated in CC tissues (P<0.05). *In vitro* experiments with silenced FABP5 showed that cell proliferation and migration were significantly decreased. In an *in vivo* xenograft model and lung metastasis model, the tumor formation ability of mice was significantly reduced (P<0.001), and tumor metastasis in each side of the lung lobe was also significantly reduced (P<0.001). The mechanism may involve the promotion of the occurrence and metastasis of CC through the upregulation of MMP-2 and MMP-9 (139). Consistent with the results of Liu et al, an experiment found that FABP5 expression was significantly upregulated in CC with lymph node metastasis, and FABP5 was an independent prognostic factor in multivariate Cox proportional risk model analysis. A Kaplan-Meier survival curve and log-rank test showed that patients with high FABP5 expression had significantly lower RFS and OS. In nude mice with lymph node metastasis, FABP5 knockdown resulted in a higher survival. Mechanistically, FABP5 expression promotes the invasion, EMT, and lymphangiogenesis by increasing the levels of intracellular fatty acids in CC to activate NF-κB signaling. Treatment with orlistat suppresses this effect (43). Previous work has suggested that high expression of FABP5 is positively correlated with the existence of lymph node metastasis in CC (44, 45). An experiment found that the long noncoding RNA LNMICC could promote the tumor growth, CC cell proliferation and lymph node metastasis by recruiting NPM1 to the promoter of FABP5 (46). Additionally, Jin et al. found that the FABP4 level in CSCC was strikingly higher than that in normal tissue (47), which is in line with the conclusions of previous investigations (140, 141). Moreover, the elevation of FABP4 has been shown to promote EMT through the activation of the AKT/GSK3β/Snail pathway in CSCC. Li et al. screened 243 genes related to lymph node metastasis in 178 TCGA CC samples and analyzed these genes by univariate and multivariate Cox regression analyses of FABP4 (HR=1.582, P < 0.001) FABP4 (HR=1.384, P=0.024). It was proven that FABP4 could be used as a prognostic factor to evaluate OS. Cell experiments also showed that FABP4 could promote the occurrence of lymph node metastasis by activating the AKT signaling pathway, thus accelerating the process of EMT (140). FABPs are undoubtedly potential biomarkers or targets in patients with CC.

## 7 Targeting the intracellular lipolytic pathway

It has been reported that intracellular lipolysis may be closely related to the survival and growth of tumor cells. Lipolysis is the process in which triglycerides stored in fat cells are hydrolyzed to produce fatty acids, thereby supplying internal or whole-body energy (142–144). This process occurs through the actions of ATGL, hormone-sensitive lipase (HSL) and monoglyceride lipase (MAGL) in turn. However, the role of lipase in cancer is still unclear. Various exceptional literature reports and reviews have also elaborated the association between lipases and cancer (144–146), among which the relationship between MAGL and cancer has been discussed the most. Castelli et al. showed that the expression of ATGL in CC was extremely high, they verified their results by bioinformatics analysis of a large human cervical cancer sample data set on an Affymetrix-U133-plus2.0 array and found that the expression level of ATGL was positively correlated with the grade of CC. Additionally, ATGL promotes tumor cell proliferation and invasion through ROS production and HIF1α induction (52). Meanwhile, HIF1α is also closely related to radiotherapy resistance and paclitaxel resistance in CC (147, 148). One study demonstrated that 2,4-dienyl-CoA reductase (DECR1) enhanced the expression of HSL to increase lipolysis and thus promote the release of fatty acids, leading to malignant progression of CC (53). Interestingly, a study also showed that the level of MAGL was upregulated in CC cells and tissues. The use of the MAGL inhibitor JZL184 or gene knockout induces apoptosis in CC cells by mediating the downregulation of Bcl-2 and the upregulation of cleaved caspase-3 and Bax (54). Lipase may be a promising target to treat CC and alleviate its drug resistance.

## 8 Targeting transcriptional regulators of fatty acid metabolism

**Figure 2:**
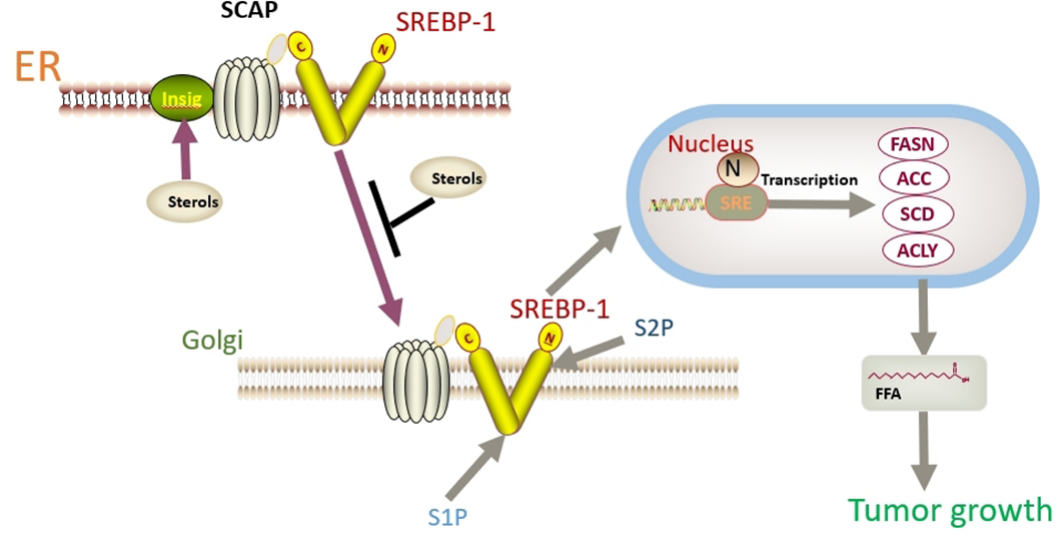
SREBP binds to SCAP in the ER and is negatively regulated by endogenous sterol levels. When sterols are abundant, INSIGs bind tightly to SCAP and restrict SREBP to the endoplasmic reticulum. Once sterol levels drop, INSIGs dissociate from the SCAP protein, and the SREBP-SCAP complex enters the Golgi. These proteins are sequentially cleaved at the Golgi by S1P and S2P. This releases the N-terminus of SREBP, which eventually binds to sterol response elements (SREs) in the nucleus to activate transcription.

**Figure 3:**
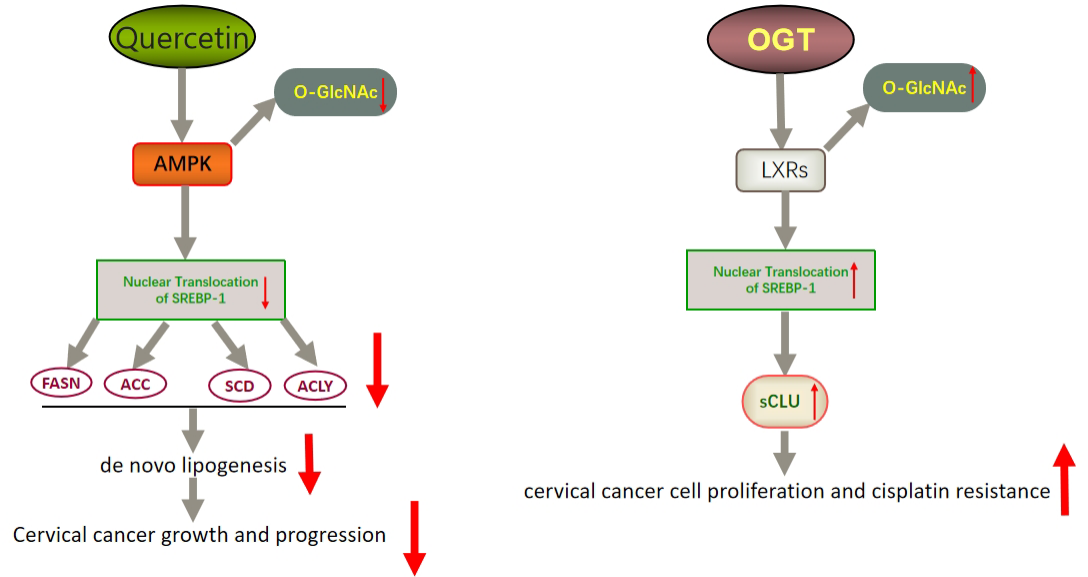
Schematic representation of quercetin- and OGT-mediated effects on adipogenesis and cell growth in CC. Quercetin could reduce the expression of SREBP-1 and its transcriptional targets by reducing the O-GlcNAcylation of AMPK. Thus, the growth of CC cells is inhibited and apoptosis is induced. OGT upregulated the expression of O-GlcNAcylated LXRs and increased sCLU levels by inducing SREBP-1 expression. Thus, CC cells become resistant to chemotherapy drugs such as cisplatin. LXRs, liver X receptors; sCLU, secretory clusterin.

In addition to FASN, another key factor worth noting is sterol regulatory element binding protein 1 (SREBP-1). SREBP-1 activation promotes the expression of FASN, ACC, and SCD1, thereby enhancing lipid synthesis (149). SREBP binds to SREBP cleavage–activating protein (SCAP) in the ER and is negatively regulated by endogenous sterol levels (150). When sterols are abundant, insulin-induced genes (INSIGs) bind tightly to SCAP and restrict SREBP to the endoplasmic reticulum. Once sterol levels drop, INSIGs dissociate from the SCAP protein, and the SREBP-SCAP complex enters the Golgi. These proteins are sequentially cleaved at the Golgi by site-1 and site-2 proteases (S1P and S2P). This releases the N-terminus of SREBP, which eventually binds to sterol response elements (SREs) in the nucleus to activate transcription (**Figure 2**) (150, 151). Several excellent reviews have elucidated the role of SREBP-1 in cancer, and tumor proliferation can be inhibited by knocking down or inhibiting SREBP-1 expression (62, 149, 152). One experiment proved that the level of SREBP-1 was high in CC cells, and quercetin (a naturally occurring polyphenolic flavonoid) could reduce the levels of SREBP-1 and its transcriptional targets by reducing the O-GlcNAcylation of AMPK. Thus, the growth of CC cells are inhibited and apoptosis is induced (**Figure 3**) (55). This is compatible with several studies suggesting that AMPK activation leads to the phosphorylation of SREBP-1, which slows cancer progression by inhibiting its nuclear translocation and the transcription of target genes (153, 154). Interestingly, O-GlcNAcylation-mediated inactivation of AMPK also accelerated the growth of colon cancer cells (155). An experiment also showed that O-linked N-acetylglucosamine transferase (OGT) upregulated the expression of O-GlcNAcylated LXRs and increased sCLU (a glycoprotein) levels by inducing SREBP-1 expression to regulate apoptosis, the cell cycle and cisplatin resistance (**Figure 3**) (56). Interestingly, Yang et al. showed that the levels of human hydroxysteroid dehydrogenase 2 (HSDL2) in CC tissues were significantly higher than those in normal tissues and HSDL2 upregulated the expression of FASN, ACSL and SREBP-1, thus inducing stronger invasiveness of CC. When SREBP-1 was knocked down, the proliferation and migration of CC cells were significantly inhibited (156). As a transcriptional regulator of lipid metabolism, SREBP-1 may become a new therapeutic breakthrough.

## 9 Targeting other lipid metabolic pathways

Lipids are a class of substances that are insoluble in water and include glycerol phosphates, triglycerides, sterols, and sphingolipids (157). A large number of studies have proved that disorders of lipid metabolism are closely related to the occurrence of various cancers. It is worth noting that the relationship between phospholipids and cholesterol levels and cancer has received much less attention in the past. However, research has shown that lysophosphatidic acid (LPA) may be a potential biomarker of gynecological cancer (65). LPA is a glycerophospholipid that stimulates cell migration and tumor cell invasion. Interestingly, Sui et al. showed that the serum LPA level in cervical cancer patients was significantly higher than that in healthy people, which was consistent with the results of Xu et al. (65), and found that LPA stimulated the progression of CC through the Ras/Raf1/MEK/ERK pathway. Noticeably, LPA could block the alterations in the caspase-3 enzyme activity caused by cisplatin, resulting in the resistance to cisplatin-induced apoptosis (57). An experiment also showed that LPA markedly reduced the expression level of the caspase-3 protein in doxorubicin hydrochloride-induced CC cells and protected CC cells from doxorubicin hydrochloride-induced apoptosis (58). These results provide sufficient experimental basis for the possibility of using LPA as a therapeutic target for CC. Lipogenesis generally includes fatty acid synthesis and the mevalonate pathway, the latter referring to isoprenoid and cholesterol synthesis. It is well known that acetyl-CoA is the raw material for the synthesis of fatty acids and cholesterol (158). Studies have shown that HMG-CoA inhibitors, statins, can not only reduce cholesterol levels but also inhibit cell proliferation and chemical resistance in gynecological cancers, including CC (159). At the same time, an experiment has found an unexpected link between fatty acid metabolism and cholesterol metabolism (160). MiR-1908 is a miRNA located in the intron of fatty acid desaturase 1 gene. It has been found that miR-1908 is highly expressed in CC, ovarian cancer, breast cancer and other tumors and is related to their poor prognosis. It is interesting that the expression of miR-1908 is regulated by free fatty acids, cholesterol, SCD1 and other factors (59). Liu et al. proved that FASN, a key enzyme in fatty acid synthesis, was highly expressed in patients with CC and found that FASN could regulate cholesterol metabolism, increase total cholesterol and free cholesterol when overexpressed, lead to lipid raft reprogramming and actin remodeling, and help enhance the invasion and migration of CC cells. After targeted inhibition of FASN, the total amount of cholesterol and free cholesterol decreased, thus effectively reducing the lymph node metastasis of CC (28). A recent experiment showed that the mechanism of FASN regulation of cholesterol metabolism in liver cancer was similar to this mechanism (161). The interference between these metabolic pathways provides more possibilities for targeted therapy of CC.

## 10 Dietary interventions

In several large studies, obesity and a high body mass index have been found to be positively associated with the development of CC (162, 163). These studies have also proved that the diet influences the occurrence and progression of cancer, and the level of fatty acid intake has been shown to contribute to these effects (164–166). Among fatty acids, omega-3 polyunsaturated fatty acids (ω-3 PUFAs), such as α-linolenic acid (α-ALA), docosahexaenoic acid (DHA), and eicosapentaenoic acid (EPA), are widely believed to have triglyceride-lowering and anti-inflammatory properties, whereas ω-6 PUFAs, including linoleic acid (LA) and arachidonic acid (AA), are thought to be involved in proinflammatory mechanisms (167–169). One study showed that increased consumption of ω-3 PUFAs (EPA) in men with prostate cancer reduced tumor vascularization and inhibited the progression of prostate cancer (170). Similarly, ω-3 PUFAs inhibit the invasion of gastric cancer through the COX-1/PGE3 signaling axis, and ω-6 PUFAs enhance the potential of malignant metastasis through COX-2/PGE2 (171). A randomized controlled study suggested that increasing the intake of ω-3 PUFAs was effective in maintaining the nutritional status and skeletal muscle mass in women with CC and alleviating the toxicity of chemoradiotherapy (172). α-ALA inhibits the growth of CC cells by downregulating the expression of HPV oncoproteins E6 and E7, thereby restoring the expression of Rb and p53 (173). Notably, DHA could induce the apoptosis of CC cells by reducing the levels of the anti-apoptotic proteins Bax, cleaved caspase-3 and Bcl-2 and regulate the levels of VEGF and MMP-9 to control the invasion of CC cells (174). However, excessive intake of ω-3 PUFAs may result in immunosuppression and other adverse effects. Since ω-3 PUFAs and ω-6 PUFAs are both essential fatty acids, it is necessary to reasonably adjust the diet, aiming to control their appropriate levels. Olive oil, a component of the Mediterranean diet, has recently been found to have antitumor effects in breast, prostate and other cancers (175). The main ingredient in olive oil is oleic acid (OA), which has recently been found to promote the progression of CC *in vitro* and *in vivo* (40, 176). However, Muhammad et al. found that monounsaturated and diunsaturated fatty acids could improve the sensitivity of obese patients with CC to radiotherapy, and the tumor volume was significantly reduced in a mouse xenograft tumor model treated with oleate and radiotherapy compared with that in the radiotherapy alone group. The supplementation of oleate and linoleate during radiotherapy increases the expression of P53, PPARγ, and CD36 to support increased free fatty acid uptake, thereby regulating the cell cycle and inducing apoptosis (177). However, these contradictory results make the effect of dietary olive oil or OA on cancer inconclusive. Complex interactions between environmental factors and genetics may partially explain this phenomenon. At a later stage, much research is still needed to explain the link between dietary olive oil or OA and cancer. Certainly, a diet combined with other treatments may have greater potential for the treatment of CC.

## 11 Conclusion

Lipid reprogramming has been widely confirmed as an important marker of CC that can act on membrane production, energy production and signal transduction to control cell growth, differentiation and motility. This review analyzed in detail the involvement of various pathways of fatty acid metabolism in CC. Targeting or knocking down proteins or enzymes involved in the process of fatty acid metabolism can effectively limit the growth and progression of CC cells, inhibit lymph node metastasis to a certain extent, improve the sensitivity of CC to chemoradiotherapy, and significantly improve the treatment effect and prognosis. Therefore, targeting fatty acid metabolism is extraordinarily attractive for the treatment of CC to achieve precise antitumor effects. However, due to the plasticity of tumor fatty acid metabolism, few preclinical studies can be effectively applied to the clinical field. A novel combination strategy or strict diet provides promising prospects. Further experiments to investigate the dynamic relationship between fatty acid metabolism reprogramming and CC and to overcome the complexity and plasticity of fatty acid metabolism in cancer are extremely important. Although this road is tortuous, fatty acid metabolism and its related lipid metabolism pathways are expected to become new targets for CC treatment.

## Author contributions

All authors contributed to the review conception and design. The first draft of the manuscript was written by PP and all authors commented on previous versions of the manuscript. All authors contributed to the article and approved the submitted version.
